# Nicotine Intervention and Communication for Empowering Reduction (NICER): study protocol for a randomized controlled trial among priority populations of people who smoke

**DOI:** 10.1186/s13063-025-09216-8

**Published:** 2025-11-17

**Authors:** Di Pei, Aubrey Juris, Prudence Nkansah, James F. Thrasher, Nicholas A. Giordano, Katherine C. Henderson, Claire A. Spears, David L. Ashley, Vanessa C. Mallory, Folashayo P. Adeniji, Jordan D. Foster, Winnie Ni, Nurul Kodriati, Adebusola Ogunnaike, Terry F. Pechacek, Alexander Kirpich, Lucy Popova

**Affiliations:** 1 Department of Health Policy and Behavioral Sciences, School of Public Health, Georgia State University, Atlanta, GA USA; 2 Nell Hodgson Woodruff School of Nursing, Emory University, Atlanta, GA USA; 3 Department of Health Promotion, Education & Behavior, Arnold School of Public Health, University of South Carolina, Columbia, SC USA; 4 TRSConsulting56, Lilburn, GA USA; 5 School of Public Health, University of Ahmad Dahlan, Yogyakarta, Indonesia; 6 Department of Population Health Sciences, School of Public Health, Georgia State University, Atlanta, GA USA

**Keywords:** Nicotine, Smoking, Health communication

## Abstract

**Background:**

Smoking is a leading cause of preventable death, disproportionately affecting people with low socioeconomic status (SES) and serious psychological distress (SPD). The U.S. Food and Drug Administration (FDA) has proposed reducing nicotine levels in cigarettes and other combusted tobacco products to minimally addictive levels, a policy with significant public health potential. However, misperceptions about very low nicotine cigarettes (VLNCs), such as beliefs that they are less harmful or ineffective for quitting, may reduce policy effectiveness. While previous randomized controlled trials (RCTs) have examined the effects of using VLNCs, none have incorporated messaging to address misperceptions. This study evaluates the impact of a messaging campaign on smoking behaviors, risk perceptions, and quit intentions among people who smoke, focusing on individuals with low SES, SPD, and neither. The primary objective is to assess whether exposure to VLNC-related messages reduces the number of cigarettes smoked per day compared to VLNC use alone. Secondary objectives include examining effects on other tobacco product use, nicotine dependence, forgoing cigarettes, perceived risks, and quit intentions.

**Methods:**

This multi-site, open-label, parallel-arm RCT will enroll 1230 adults who smoke (*n* = 410 per group: with SPD, low SES, neither category). After a 1-week baseline period, participants will be randomized (1:1) to receive either (1) VLNCs with messaging or (2) VLNCs only (control). Messaging will include pack inserts and digital ads shown during weekly visits to address misperceptions and encourage quitting. Participants will complete daily logs via text messages and attend weekly visits over 4 weeks for data collection, including self-reported smoking behavior, expired carbon monoxide (CO) samples, and questionnaire assessments. The primary outcome is the number of cigarettes smoked per day in the final study week (week 4). Secondary outcomes include the use of other tobacco products, nicotine dependence, forgoing cigarettes, and quit intentions.

**Discussion:**

This trial will be the first to examine the effects of a messaging campaign accompanying VLNC use among priority populations. Results will inform FDA regulatory strategies and public health messaging to support nicotine reduction policy implementation.

**Trial registration:**

ClinicalTrials.gov NCT06787937. Registered on 22 January 2025.

## Administrative information {1b}

**Table:** 

Item	Description
Primary Registry and Trial Identifying Number {4}	ClinicalTrials.gov, NCT06787937. Registered 22 January 2025. (URL: https://clinicaltrials.gov/study/NCT06787937?term=NCT06787937&rank=1)
Secondary Identifying Numbers	n/a The trial is registered only at ClinicalTrials.gov; no additional registry numbers.
Source(s) of Monetary or Material Support	Research reported in this publication was supported by the National Cancer Institute of the National Institutes of Health and the Food and Drug Administration Center for Tobacco Products (R01CA239308).
Primary Sponsor and contact information {3b}	Georgia State University. PO Box 3999, Atlanta, GA 30302-3999
Role of sponsor and funder {3c}	The content is solely the responsibility of the authors and does not necessarily represent the official views of the National Institutes of Health or the Food and Drug Administration. The funders had no role in study design, data collection and analysis, decision to publish, or preparation of the manuscript.
Contact for Public Queries	Lucy Popova, PhD, Telephone: 404–413-9338; E-mail:lpopova1@gsu.edu
Contact for Scientific Queries	Lucy Popova, PhD, Telephone: 404–413-9338; E-mail:lpopova1@gsu.edu
Public Title	Nicotine intervention and communication for empowering reduction among priority populations of people who smoke (the NICER study)
Scientific title	The NICER Study (Nicotine Intervention and Communication for Empowering Reduction) protocol: a randomized controlled trial evaluating effects of messages about reduced nicotine cigarettes and policy
Countries of Recruitment	United States
Health Condition(s) or Problem(s) Studied	Smoking
Intervention(s)	Message campaign
Key Inclusion and Exclusion Criteria	Inclusion criteria: Adults aged ≥ 21 years who self-report having smoked ≥ 100 cigarettes in their lifetime and currently smoke on ≥ 27 of the past 30 days; expired breath carbon monoxide (CO) ≥ 6 ppm confirming regular smoking; able to speak, read, and write in English; available for the study period; and willing to use the study cigarettes. Exclusion criteria: Current pregnancy or lactation, or intent to become pregnant within the next 2 months; living in the same household as an enrolled participant; or unwillingness to use the study cigarettes as part of the trial.
Study Type	This is a multi-site, parallel-group, open-label randomized controlled trial with a 1:1 allocation to the treatment condition and control condition.
Date of First Enrollment	First enrollment began 11 September 2025
Sample Size	1,230
Primary outcome(s)	Cigarettes smoked per day
Key Secondary outcome(s)	Use of other tobacco products; Use of nicotine replacement therapy; Perceived risks of smoking VLNCs; Quit intention; Nicotine dependence; Forgoing cigarette behaviors.
Ethics Review	This study was approved by the GSU institutional review board (protocol H25318).
Individual Trial Participant Data sharing statement	All study data will be deposited in the ScholarWorks@Georgia State University institutional repository within 1 year of study completion. The compiled dataset will be publicly accessible through the ScholarWorks@Georgia State University website. Users seeking access must adhere to specific conditions as specified by the ScholarWorks. Redistribution of the data to third parties will not be permitted.

**Protocol version {2}**

 October 2025. Version 4

## Introduction

### Background and rationale {9a}

Smoking remains the leading cause of preventable death and disease in the US [1, 2]. Tobacco-related health disparities endure, with smoking concentrated in socially and economically disadvantaged populations. Although most people who smoke in the US want to quit, only about 7% successfully quit each year [3], with even lower success rates among people with mental health conditions and those with low socioeconomic status (SES) [3–6].

To address this public health crisis, the FDA has issued a proposed rule to reduce nicotine in cigarettes and certain other combusted tobacco products (cigarette tobacco, roll-your-own tobacco, cigars, and pipe tobacco) to minimally addictive levels [7]. This groundbreaking strategy can potentially save millions of lives by increasing cessation rates and preventing people who experiment with cigarettes from developing an addiction and moving to regular smoking [8]. Many randomized clinical trials (RCTs) have found that very low nicotine cigarettes (VLNCs) are less addictive than regular cigarettes, reduce the number of cigarettes smoked per day, and increase quit attempts [9–11]. Studies with priority populations, including people with mental health conditions and those from low SES backgrounds, found that VLNCs reduce smoking behavior and nicotine dependence in these groups to a similar extent as in the general population, reinforcing the general applicability of the policy [12–18].

However, these RCTs evaluating the effects of smoking VLNCs did not incorporate messages about the VLNC products or the reduced nicotine policy; frequently, participants were blinded to the fact that they were smoking VLNCs. When the reduced nicotine policy is implemented in the real world, it will be publicized. Without appropriate educational messages, misinformation and misperceptions about the reduced nicotine cigarettes and the policy might proliferate, spurring backlash and ultimately reducing the potential effectiveness of the policy. Past studies documented various misperceptions about VLNCs, including the notion that they are less harmful than regular cigarettes [19–22], that they make people smoke more [23], appeal to people who formerly smoked, and are a conspiracy between the FDA and the tobacco companies to keep people hooked on nicotine via alternative delivery methods [24]. Therefore, implementing the reduced nicotine policy will require accompanying communication strategies that address misperceptions about VLNCs and the policy itself.

Messages correcting misperceptions around VLNCs may have a meaningful impact on perceived risk and behaviors. Past studies developed and tested the effects of messages on perceptions related to VLNCs and/or the policy, utilizing either textual statements or fully developed advertisements [25–30]. These studies suggest certain statements or ads can effectively reduce misperceptions. However, the messaging studies were mostly hypothetical, without participants actively using VLNCs. This study will take the next step by bringing together two previously separate areas of research: studies examining the behavioral effects of VLNCs use through RCTs, and those testing the effects of messages on correcting misperceptions about VLNCs in hypothetical scenarios. By assessing the effects of messages on people who smoke while they are using VLNCs, this study will provide important evidence to inform FDA regulatory actions around product review, product standards, and public education on nicotine.

### Explanation for the choice of comparator {9b}

Participants in both the intervention and control conditions will receive VLNCs at no cost. The control condition, which does not include the message intervention, serves as a comparator to isolate the independent effects of messaging on study outcomes.

### Objectives {10}

The primary objective of the study is to examine the effect of messages about VLNCs accompanied by VLNC use (vs. use of VLNCs without messages) on the number of cigarettes smoked per day among people who smoke from three groups: those with past-month serious psychological distress (SPD), those with low SES, and those in neither category. The secondary objectives are (1) to examine the effect of messages about VLNCs in combination with VLNC use (vs. use of VLNCs without messages) on the use of other tobacco products (e.g., cigarillos, e-cigarettes), cigarette dependence, and cessation-related outcomes over the study period, and (2) to examine the effect of messages about VLNCs in combination with VLNC use (vs. use of VLNCs without messages) on perceptions and behavioral intentions.

## Methods: patient and public involvement and trial design

### Patient and public involvement {11}

There was no formal patient or public involvement in the design, conduct, or reporting of the trial. However, the team will engage with the public through (a) community partnerships and healthcare-system outreach used in recruitment, particularly to reach racially/ethnically diverse smokers with SPD and low SES, and (b) planned dissemination of findings to public health agencies, tobacco control organizations, policymakers, and the broader public.

### Trial design {12}

This randomized, open-label, controlled, multi-site study will test the efficacy of a messaging campaign about reduced nicotine cigarettes and policy in combination with the use of VLNCs. People who smoke will come from three populations: those with past-month SPD, those with low SES, and those in neither category (*n* = 262 completes for each group). Participants will complete a 1-week baseline followed by a 4-week study. They will be randomized (1:1 allocation) to either (1) the treatment condition, where VLNCs are provided during weekly visits and participants are exposed to messages about VLNCs and reduced nicotine policy, or (2) the control condition, where VLNCs without messages are provided during weekly visits. Participants can select menthol or non-menthol cigarettes based on preference. Data will be collected during in-person weekly visits and daily logs throughout the duration of the study.

**Fig. 1:**
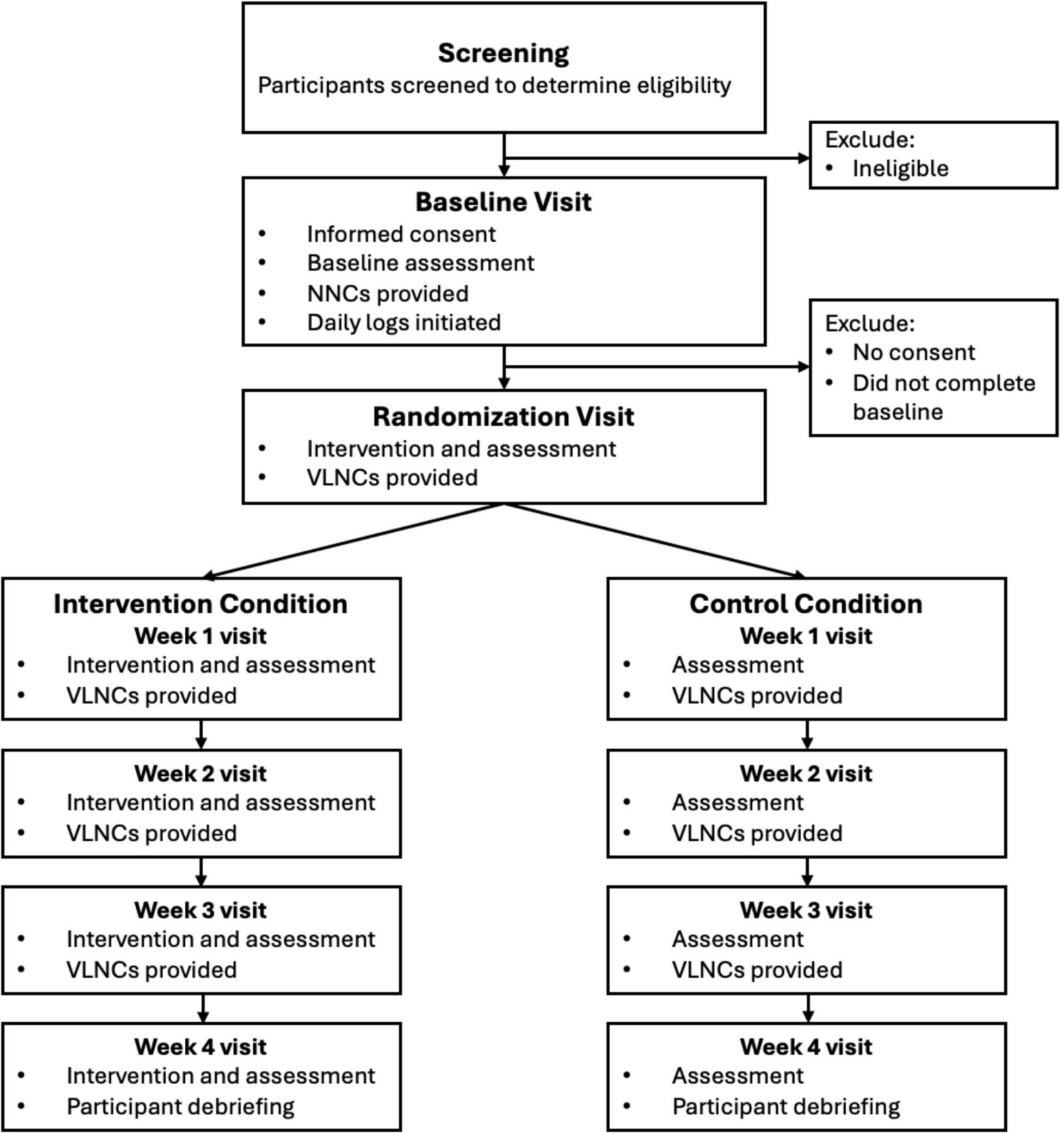
Participant flow. NNCs, normal nicotine content cigarettes; VLNCs, very low nicotine content cigarettes

Figure 1 illustrates the study design and participant progression. After screening, eligible participants will schedule a baseline visit at their preferred site, where they will complete baseline questionnaires, provide expired carbon monoxide (CO) samples, and receive a 1-week supply of the Spectrum Normal Nicotine Content Cigarettes (NNCs). One week after the baseline visit, participants will return for their randomization visit, where they will be randomly assigned into either the treatment condition (VLNCs + messages) or the control condition (VLNCs only). They will then attend 4 weekly visits over the following weeks. During each weekly visit, participants will complete questionnaires, provide an expired CO sample, and receive a 14-day supply of VLNCs (with or without the inserts, according to the assigned condition). Daily logs will be collected via text messages (sent via Twilio) with links to brief online surveys in Research Electronic Data Capture (REDCap), sent each day at a time the participant chooses, from the baseline period through week 4.

### Screening and baseline visit

#### Screening

Individuals interested in participation will leave their contact information on a voicemail or on a form on our website. Research staff will call each participant and describe the study in detail. After obtaining verbal consent, the participants will be asked questions over the phone to determine initial eligibility. If eligible and interested, they will be scheduled for an in-person baseline visit.

#### Baseline visit

Participants will attend their baseline visit at the study site that they select. Staff will provide a detailed study description, answer questions, finalize eligibility, and obtain written informed consent. Participants will bring packages of their usual cigarette brand to document brand preferences, complete questionnaires, and provide expired CO samples. They will receive a 1-week supply (at 150% of their normal smoking rate) of NNCs and be instructed to bring their entire supply (empty packs and unused cigarettes) to the randomization visit. Participants will be given two handouts: one graphically explaining the study flow and another explaining the use of tobacco products during the study: (1) that they are asked to only use study cigarettes; (2) if they use regular cigarettes, to report them; (3) they can use other tobacco products as they would like, but with a reminder that combusted tobacco products are the most harmful, and non-combusted less harmful, with nicotine replacement therapy (NRT) being the lowest harm. Participants who do not own a mobile phone will be given one for the duration of the study. Study personnel will explain the text messaging procedures and send a test message to the participant, who will respond and ask any questions. During the week following the baseline visit, participants will use the Spectrum NNCs and complete daily logs.

### Randomization, intervention and weekly visits

During the randomization visit (1 week after baseline), participants will be randomized into treatment (VLNCs + messages) or control (VLNCs only). Participants will be randomized using a 1:1 ratio into treatment (VLNCs + messages) or control (VLNCs only). We will use simple randomization with stratification by site, readiness to quit (planning to quit in the next 6 months vs. not), and menthol preference (menthol vs. non-menthol) with no required sample size for any of these strata. During the randomization and weekly visits, participants will complete questionnaires and provide an expired CO sample. At each visit, they will receive a 14-day supply of VLNCs (with or without the inserts, according to the assigned condition). This will ensure adequate availability of cigarettes in the different locations where participants may typically keep a supply (home, work, vehicle, etc.) as well as avoid expending the entire supply if they miss a scheduled visit. They will be instructed to return all unused cigarettes and empty cigarette packs each week; empty packs will be saved, and unused packs will be redistributed to the participants during subsequent visits. At each visit, they will see 4 messages about VLNCs (in the treatment condition) or, in the control condition, no messages. At each visit, participants will be counseled about daily log completion (based on staff review of compliance during the prior week), visit attendance, and returning unused cigarettes. Throughout the 4 weeks of the study, participants will continue to complete daily logs. During the last visit (end of week 4), participants will be referred to smokefree.gov and given a Quitline number.

### Daily logs

From the baseline period through week 4, daily logs will be collected via text messages (sent via Twilio) with links to brief online surveys in REDCap. Daily logs will assess their use of study and non-study cigarettes and other combusted and non-combusted tobacco products. Participants will report tobacco use in the past day.

## Methods: participants, interventions and outcomes

### Trial setting {13}

Recruitment and in-person visits will be administered at multiple sites: Georgia State University (PI Popova) and Emory University in partnership with Grady Memorial Hospital (Co-I Giordano). Participants will be allowed to select which location they prefer for their in-person visits (GSU or Emory University; Grady Memorial Hospital is only a recruitment site). Study procedures and training will be standardized based on the protocol.

### Eligibility criteria for participants {14a}

Eligibility criteria are presented in Table 1.

**Table 1:** Inclusion and exclusion criteria

**Inclusion**	
Age	≥ 21
Smoking status	Self-report as having smoked 100 cigarettes in their lifetime and currently smoking on 27+ days out of past 30, expired breath carbon monoxide (CO) ≥ 6 ppm [31] to assess regular smoking
Comprehension	Able to speak, read, and write in English
Availability	Available for period of study
Study cigarettes	Willingness to try novel research cigarettes
**Exclusion**	
Pregnancy	Current pregnancy or lactation; intention to become pregnant in the next 2 months
Household	Member of the same household as a study subject
Study cigarettes	Unwilling to use research cigarettes as part of the trial

### Eligibility criteria for sites and those delivering interventions {14b}

#### Sites

In-person visits are conducted at Georgia State University and Emory University (in partnership with Grady Memorial Hospital); Grady serves as a recruitment site only. Study procedures are standardized across sites per protocol.

### Individuals delivering interventions

Site PIs and project coordinators will oversee protocol adherence. All research personnel are trained in study procedures, human subjects protection, and regulatory requirements prior to site initiation. Standard operating procedures are used to ensure consistent delivery and data collection, with regular cross-site meetings for quality control.

### Who will take informed consent? {32a}

During the baseline visit, research staff at all sites will provide potential participants with a copy of the informed consent form (ICF), review the ICF in detail, and give potential participants ample time to ask questions and discuss their thoughts about possible research participation. For eligible individuals who are interested in participating, the ICF will be signed and dated by the participant and staff member. A copy of the ICF will be given to the participants.

### Additional consent provisions for collection and use of participant data and biological specimens {32b}

N/a—no biological specimens will be collected.

This study does not involve the collection, storage, or use of biological specimens. Specifically, while we will assess expired CO levels through a non-invasive breath test, no physical samples will be collected, retained, or stored. CO breath measurements are taken in real-time using a portable monitor and are recorded as numerical values only. Since no biological specimens (e.g., blood, saliva, urine, or tissue) are obtained, no additional consent provisions for biospecimen collection or use are necessary.

## Intervention and comparator

### Intervention and comparator description {15a}

#### Very low nicotine cigarettes (treatment and control conditions)

VLNCs are cigarettes with much lower levels of nicotine than normal cigarettes (possibly below addictive levels). In this study, we will use nicotine research cigarettes (NRCs) from the National Institute on Drug Abuse Drug Supply Program (NIDA DSP). Study cigarettes will be obtained from NIDA for free (NOT-DA-14-004). These will be Spectrum investigational cigarettes: regular (NRC102) and menthol (NRC103) VLNCs, which contain approximately 0.4 mg nicotine per g of tobacco. For the 1-week baseline, we will provide participants with normal nicotine cigarettes from NIDA (15.8 mg/g), regular (NRC600) or menthol (NRC601).

### Messages (treatment condition only)

Messages about VLNCs and reduced nicotine policy will be professionally developed as full-color inserts (4), still ads (4), and videos (4) in collaboration with a social marketing company using the results of our previous studies. Both general population messages and messages tailored to specific groups (people who smoke with SPD, low SES) developed in previous focus groups will be used. Messages will focus on key themes: (1) VLNCs still contain harmful chemicals and cause death and disease; (2) lower nicotine makes it easier to quit; (3) benefits of quitting smoking; 4. Reducing the nicotine level by 95% is an opportunity to quit. We will have 2 tailored messages for each priority population. For example, messages tailored for the SPD group will focus on the negative impact of smoking on anxiety, while messages for the low SES group will focus on the costs of smoking. Additional themes emerging from focus groups may also be used.

Messages will be delivered as inserts inside the packs and ads shown to the participants during the in-person study visits (4 ads at each of 5 weekly visits). Ads will be embedded in the REDCap online questionnaires and shown on a computer monitor or iPad. Pack-a-day smokers will have about 48 exposures over a 4-week study period, including exposure to 4 ads during each weekly visit (totaling 20 exposures) and daily exposure to 1 insert message (totaling 28 times). With this design, we are approximating the intensity of the real-world communication campaign.

### Criteria for discontinuing or modifying allocated intervention/comparator {15b}

At each weekly visit, participants will be asked if they have made a quit attempt in the past week.

If a participant has made a quit attempt in the past week, research staff will:

- Encourage the participant to continue abstaining from smoking.
- Schedule normal weekly visits.
- Provide the participant with the tobacco quitline number.
- Offer the option to either take home the study product or decline it. If the participant chooses to take home the study product, recommend they store the product out of sight to avoid smoking triggers.

If the participant declines the study product, instruct them to contact the research coordinator if they lapse and would like to pick up the product before the next visit.

For the safety concerns, participants will be withdrawn immediately from the study if any of the following occur:

- Cardiovascular disease (CVD) event: Typically includes MI (heart attack), PTCA (angioplasty/stenting), bypass surgery, stroke, peripheral vascular disease (arterial blockages in arms or legs leading to procedure or surgery). Less common CVD problems would be new cardiac arrhythmias (e.g., new atrial fibrillation) or new valvular disease (e.g., mitral or aortic regurgitation).
- DVT/PE (deep vein thrombosis/pulmonary embolism, i.e., blood clots in the venous system).
- Psychiatric Hospitalization: A subject will be withdrawn if they are hospitalized for psychiatric reasons at any time during participation in the study.
- Pregnancy: If subject indicates they are pregnant or have a positive pregnancy test after receipt of study products, they will be withdrawn from the study if they are continuing to use any tobacco product, and the pregnancy will be documented as an adverse event that will remain open until delivery. At that time the research staff will contact the participant to ask a few questions about the baby’s health and will update the open Adverse Event.
- Expired breath CO increase: A subject will be withdrawn from the study if the average of the 2 CO readings is 100 ppm or greater.

The following will be monitored and can lead to withdrawal by the PI:

- Expired breath CO increase: An adverse event will be documented if the average of the 2 (or 3) CO readings is:
  o CO is greater than 50 ppm if CO at baseline is < 20 ppm.
  o CO is greater than 60 ppm if CO at baseline is 20–34 ppm.
  o CO is greater than 70 ppm if CO at baseline 35–49 ppm.
  o CO is greater than 80 ppm if CO at baseline 50–64 ppm.
  o CO is greater than 90 ppm if CO at baseline 65–80 ppm.
- Changes in tobacco product use: if during the weekly visits, participants self-report a greater than 100% increase in cigarette per day (total cigarettes, including study and non-study cigarettes) relative to baseline, or a 50% increase in cigarettes per day relative to baseline that is accompanied by daily use of alternative nicotine products.
- Any hospitalization or debilitation in which participation in the study could be detrimental to the recovery process. This will be self-reported by the subject and will be reviewed by the PI to determine whether continued participation in the study is appropriate.
- If a participant is behaving in an inappropriate or threatening manner, admits to lying about eligibility criteria, including omitting previous medical diagnoses and medications, is participating in other smoking research studies that could affect the primary outcome measures, does not follow study instructions, etc., then the PI can withdraw him/her from the study at the PI’s discretion.
- If a participant fails to attend randomization visit, they may not be eligible to reschedule this visit or continue participation in the study without PI approval.
- If there is reason to believe the participant is sharing large quantities of the study product with other people.

### Strategies to improve adherence to intervention/comparator {15c}

Study products and procedure compliance will be monitored during weekly visits. Participants will be encouraged to discuss any concerns or obstacles associated with the use of the study products. Research staff will emphasize the importance of honest reporting to participants. Subjects will be told that it is crucial for them to report on the use of non-study products, even though it is discouraged. If participants experience difficulties using only study products (e.g., due to taste, withdrawal symptoms), staff will explore the underlying reasons and collaborate with them to develop strategies for overcoming these challenges while adhering to the protocol requirements.

### Concomitant care permitted or prohibited during the trial {15d}

All participants will be provided a handout of Community Health Resources, which include contact information for resources for connecting with behavioral health clinicians to diagnose and manage anxiety and depressive symptoms, as well as information on low-cost medical clinics, housing, and food insecurity. These resources will also include the 24-h intake hotline at Grady Behavioral Health and links provided by the Fulton County Department of Public Health for connecting residents with affordable behavioral health care. Participants recruited at all sites will be instructed to contact their clinician for additional counseling based on Kessler-6 scores indicative of elevated SPD symptom severity [32]. The study coordinator will immediately contact hospital security and clinic staff via the Grady internal hotline to walk any person screened on site expressing self-harm intentions to the emergency department for evaluation.

### Ancillary and post-trial care {34}

During the last visit (end of week 4), participants will be referred to smokefree.gov and given a Tobacco Quitline number.

### Outcomes {16}

#### Primary outcome

The primary outcome is the average number of cigarettes smoked per day (study and non-study cigarettes) based on 7 days’ daily logs submitted through text message prompted surveys during the last week of the trial (before the week 4 visit).

### Secondary outcomes

The following outcomes will be based on 7 days’ daily logs submitted through text message prompted surveys during the last week of the trial (before the week 4 visit). Use of other combusted (e.g., little cigar and cigarillos) and noncombusted (e.g., e-cigarettes) products will be assessed by asking participants to enter an integer indicating the number of each product used per day. Use of nicotine replacement therapy (NRT) or prescription cessation medications will be assessed using a multiple-choice question, allowing participants to select all NRT or prescription cessation medications used from a provided list of options.

The following outcomes will be based on a questionnaire completed during the week 4 visit. Perceived risks of smoking VLNCs will be measured by asking participants’ perceived likelihood of overall harm to health under conditions of smoking VLNCs [33]. Response options range from 1 (Not at all likely) to 5 (Extremely likely), with higher scores representing a better outcome. An additional option, “Don’t know,” is also provided. Self-efficacy to quit smoking will be measured with multiple items, assessing perceptions about the strength of confidence in one’s ability to quit smoking [34]. Response options range from 1 (not at all) to 7 (extremely), with higher scores representing a better outcome. An additional option, “Don’t know,” is also provided. Nicotine dependence will be assessed using the Fagerstrom Test for Nicotine Dependence scale, a six-item questionnaire measuring the quantity of cigarette consumption, the compulsion to use, and dependence [35].

Forgoing cigarettes will be assessed using daily logs submitted throughout the 4 weeks of the trial, with two items asking whether participants chose to skip cigarettes they normally would have smoked and whether they stubbed/butted out a cigarete before finishing it [34, 36]. Response options are as follows: 1 (no), 2 (yes, once), and 3 (yes, several times), with higher scores representing a better outcome. Quit intention will be measured with multiple items asking participants’ intention and motivation to quit in the next month and 6 months [37]. Response options range from 1 (not at all) to 7 (extremely), with higher scores indicating a strong intention to quit. An additional option, “Don’t know,” is also provided.

At weekly visits post randomization, participants will be asked to complete a cigarette evaluation of VLNCs, where participants recall the last time they smoked the study cigarettes and rate subjective effects of the study cigarettes: good taste, satisfaction, dizziness, reduced appetite, nausea, reduced cravings, and enjoyable sensations in the throat and chest at the week 4 visit [38]. Expired breath CO will be measured using Micro + Smokerlyzer™.

#### ***Hypotheses***

With the primary aim of testing the effect of messages about VLNCs accompanied with VLNC use (vs. use of VLNCs without messages) on the number of cigarettes smoked per day, we hypothesize that participants exposed to messages in combination with VLNC use will smoke fewer study and non-study cigarettes per day compared to those using VLNCs without receiving any messages.

The second aim is to examine the effect of messages about VLNCs in combination with VLNC use (vs. use of VLNCs without messages) on the use of other tobacco products (e.g., cigarillos, e-cigarettes), cigarette dependence, and cessation-related outcomes over the study period. We hypothesize that exposure to messages in combination with VLNC use will lead to (1) less non-study cigarette use, (2) less cigarette dependence, and (3) an increase in the forgoing of cigarette behaviors compared to VLNCs without messages. The third aim is to examine the effect of messages about VLNCs in combination with VLNC use (vs. use of VLNCs without messages) on perceptions and behavioral intentions. We hypothesize that exposure to messages in combination with VLNC use will lead to (1) increased perceived risk of smoking VLNCs, (2) increased self-efficacy to quit smoking, and (3) increased intention to quit or cut down smoking compared to VLNCs without messages.

### Harms {17}

An adverse event (AE) is defined as any unfavorable and unintended diagnosis, symptom, sign, syndrome, or disease which either occurs during the study, having been absent at baseline, or if present at baseline, appears to worsen. Adverse events are to be recorded regardless of their relationship to the study intervention. A serious adverse event (SAE) is defined as any untoward medical occurrence that results in death, is life threatening, requires inpatient hospitalization or prolongation of existing hospitalization, results in persistent or significant disability/incapacity, or is a congenital anomaly. The study coordinator at each site will conduct daily oversight of participant safety and will meet regularly with the PI to review progress and discuss any problems or concerns. Adverse events that are not deemed “serious adverse events” will be included in the final report to the investigators, Institutional Review Board (IRB), and National Cancer Institute (NCI).

SAEs that are unanticipated, serious, and possibly related to the study intervention will be reported to the IRB and NCI in accordance with requirements. Georgia State University defines an unanticipated event as an event that was (1) unforeseen; (2) more likely than not related to the research; and (3) either caused harm to participants or others or placed them at increased risk of harm. Unanticipated events include any harm or injury (physical, psychological, social, or economic) or other unexpected events occurring during the course of participation in a research study. As required by the GSU IRB, the PI will report a summary of each unanticipated event to the IRB using the IRB Unanticipated Event form within 7 business days.

Unexpected fatal or life-threatening SAEs related to the intervention will also be reported to the NCI Program Officer and investigators within 7 days. Other serious and unexpected AEs related to the intervention will be reported to the NCI Program Official within 15 days. Anticipated or unrelated SAEs will be handled in a less urgent manner but will be reported to the IRB and NCI.

### Participant timeline {18}

A participant’s direct study involvement is 6 weeks, including 1 week of screening, 1 week of baseline, and 4 weeks of the trial (randomization and weekly visits). There may be some variation due to scheduling constraints. The duration of the study recruitment period will be approximately 48 months. The duration to complete all study procedures and initial data analysis is estimated to be 60 months (48 months to complete the intervention and weekly visits; an additional 6 months for primary data analysis and an additional 6 months for other analysis). Study assessments and schedule are outlined in Table 2.

**Table 2:** Schedule of assessments

Variable/timepoint	Baseline visit	Randomization visit	Weekly visit 1–3	Weekly visit 4	Daily log (online)
**Outcomes**					
Cigarettes per day	x				x
Use of other tobacco products	x				x
Use of cessation medications	x				x
Forgoing cigarettes					x
Quit attempts	x	x	x	x	
Perceived risk of VLNCs	x	x	x	x	
Self-efficacy	x	x	x	x	
Quit intention	x	x	x	x	
Nicotine dependence	x	x	x	x	
Cigarette evaluation	x	x	x	x	
Carbon monoxide	x	x	x	x	
**Individual characteristics**					
Demographics	x				
Smoking status	x				
Past quitting	x				
Other tobacco products use	x				
SPD	x	x	x	x	
Pre-existing readiness to quit	x				
**Health monitoring**					
Alcohol and drug use	x	x	x	x	
Health changes		x	x	x	
Respiratory health	x	x	x	x	

### Sample size {19}

In the randomized clinical trial, participants will be randomized into one of two conditions: treatment (VLNCs with messages) or control (VLNCs only). We will conduct three separate trials, each targeting a specific group of people who smoke: (1) individuals with serious psychological distress (SPD), (2) individuals with low SES (defined as having a high school education or less or with household income less than 200% of the federal poverty level), and (3) individuals who are neither of those two groups. For each group, we will have 262 participants to complete the 4-week study. This number accounts for a 20% attrition during the 1-week baseline and an additional 20% attrition during the study period; thus, we will enroll 410 participants and randomize 328 participants to achieve 262 completes for each smoker group. The projected attrition rates are conservative and are based on our own studies and past RCTs with VLNCs [39–41], including with the same priority populations (e.g., 24% attrition during an intensive 8-week study that used ecological momentary assessments in the similar populations in Atlanta, GA [42]; 18% attrition for a 12-week study with smokers with low SES and mental health conditions [13]).

Based on the above assumptions and adjusting for missing data (0–20%) in daily logs, with 262 participants in each participant group completing our study, we will be able to detect the following effect sizes. For data collected in daily logs (28 observations per person): primary outcome (cigarettes per day) and secondary outcomes (use of other tobacco products, cessation medications, and foregoing cigarettes), the study has sufficient (0.80) power to detect small effect sizes (*d* = 0.11–0.17, depending on the level of autocorrelations and missing data). For data collected in weekly in-person visits (5 observations per person): secondary outcomes related to cessation behaviors, perceptions, and physiological responses, the study has sufficient power to detect small-to-medium effect sizes (*d* = 0.27–0.38, depending on the level of autocorrelations and missing data). Cohen’s definition of effect sizes will be used (small *d* = 0.20, medium *d* = 0.50, large *d* = 0.80 [43, 44]).

For binary outcomes, such as making a quit attempt, we will be able to detect odds ratios (OR) between 1.27 and 1.41 for daily log data (28 observations per person, depending on the level of autocorrelations and missing data) and OR = 1.74–2.22 for weekly data (5 observations per person). Past research found baseline values of 35% for the proportion of participants who tried to quit using VLNCs [41]. We will be able to detect small effect sizes (small OR = 1.68, medium OR = 3.47, large OR = 6.71 [45]).

Overall, the study is sufficiently powered to detect small effect sizes, which is necessary based on past research that found that messaging campaigns have at most small effects [46, 47].

### Recruitment {20}

The study has a comprehensive, multipronged recruitment strategy that focuses on media, community outreach, and strong partnerships with local healthcare systems and community partners, with targeted recruitment of racially/ethnically diverse smokers with SPD and low SES in Atlanta, GA.

At GSU, participants will be recruited through flyers (posted at venues including local community health centers, near MARTA and bus stops, community centers and shelters, etc.) and media (e.g., radio; print media in local newspapers and buses/trains). We will stimulate recruitment by giving presentations, talking with staff, providing study information, and supplying flyers at local clinics and community organizations. Participants will also be recruited through online sources (e.g., Craigslist) and by word of mouth. Individuals interested in participation will leave their contact information on a voicemail or on a form on our website. GSU research staff will call each participant and describe the study in detail. After obtaining verbal consent, the participants will be asked questions over the phone to determine initial eligibility. If eligible and interested, they will be scheduled for an in-person baseline visit.

In addition, at Grady and Emory, participants will be recruited through flyers placed in clinics and distributed by clinician partners, which will include a link to the screening questionnaire. The self-reported smoking status and other eligibility criteria of potential participants will be confirmed by a research coordinator via phone. Those who are eligible and interested will be scheduled for an in-person baseline visit.

#### Assignment of interventions: randomization

### Sequence generation: who will generate the sequence {21a}

During the randomization visit (after 1-week baseline), a separate computer-generated allocation sequence will be created for participants at each site using stratified randomization to ensure balance across conditions.

### Sequence generation: type of randomisation {21b}

Participates will be stratified by readiness to quit (planning to quit in the next 6 months vs. not) and menthol preference (menthol vs. non-menthol). Within each stratum, participants will be randomized in a 1:1 ratio to either the treatment (VLNCs + messages) or the control (VLNCs only) condition.

### Allocation concealment mechanism {22}

To ensure allocation concealment, randomization results will be stored in REDCap, accessible only to the research team members responsible for allocation.

### Implementation {23}

A research coordinator at each site will work with the PI, Co-I, and clinical staff at Grady to enroll participants and assign participants to the trial conditions.

## Assignment of interventions: blinding

### Who will be blinded {24a}

Trial participants will be blinded to the study hypotheses. They will be informed that they will be randomized to view (or not) campaign messages related to VLNCs.

### How will be blinding be achieved {24b}

Blinding is limited to concealing the study hypotheses from participants. Consent materials state only that participants may or may not view VLNC-related messages, without specifying hypotheses about expected effects.

### Procedure for unblinding if needed {24c}

N/a. There are no planned circumstances under which unblinding would be required, as the nature of the message intervention does not pose risks necessitating disclosure of group assignment.

## Data collection and management

### Plans for assessment and collection of outcomes {25a}

Data will be obtained via participant self-report surveys during in-person visits (collected using the REDCap system) and through daily logs (using the Twilio text messaging integration with REDCap), in addition to biochemical testing (expired CO for biochemical verification of smoking status) during in-person visits.

Standard operating procedures will be applied to each visit and procedure to ensure consistency. PI and Co-Is will train research staff and ensure quality control throughout the study, where the protocol and procedures will be carefully described. Case Report Form books will be created to maximize parallel recording of data across studies. Each visit will have a checklist of all the measures that need to be administered and the order in which these measures are administered. Additionally, online meetings will be held regularly with the lead site PIs and coordinators.

### Plans to promote participant retention and complete follow-up {25b}

Participants will receive financial compensation for the time and inconvenience associated with participation: $50 for each in-person visit, $10 per visit to defray the cost of travel; and $5 for each completed daily log, as past research shows that compensating for each assessment (and reminding participants of that) increases retention [48]. Participants will receive cash compensation at each weekly visit. If participants withdraw from the study or do not complete certain parts, they will be compensated for the portions they complete. If they are unable to attend the final in-person visit, they have the option to receive a gift card in the mail. The maximum compensation is $535/person.

### Data management {26}

Questionnaire data will be collected and managed using the REDCap (Research Electronic Data Capture) system available at Georgia State University. This will provide a secure way to collect and manage data while avoiding potential issues with manual data entry. Georgia State University and Emory (Co-I Giordano) belong to a consortium of institutional partners that work to maintain a software toolset and workflow methodology for electronic collection and management of research data. REDCap data collection projects rely on a study-specific data dictionary defined by the research team. The research team can create and design surveys in a web browser and engage potential respondents using a variety of notification methods. Both REDCap and REDCap Survey systems provide secure, web-based applications that are flexible enough to be used for a variety of types of research, provide an intuitive interface for users to enter data, and have real-time validation rules (with automated data type and range checks) at the time of entry. These systems offer easy data manipulation with audit trails and reporting for monitoring, reporting, and querying patient records, and an automated export mechanism to common statistical packages (SPSS, SAS, Stata, R/S-Plus). The REDCap application is securely housed in Amazon AWS and managed by the Research Solutions department at Georgia State University. All web-based information transmission is encrypted, and the data are encrypted at rest. Amazon AWS’s data centers are state-of-the-art, nondescript, and secure facilities. Data will be downloaded from REDCap into a secure server that is managed by Georgia State University or Emory, password-protected, and available only to research personnel who have completed training in research with human subjects.

### Confidentiality {33}

All of the materials collected are for research purposes only, and data will be kept in strict confidence. Confidentiality will be ensured by the use of identification codes. All data collected will be identified with an alphanumeric identification code unique to the participant. Records that link each participant to their identifier will be kept locked in secure computer files at the sites coordinating data collection (GSU, Emory, and Grady), and only the PI and the project manager will have access to the list of identifiers.

## Statistical methods

### Statistical methods for primary and secondary outcomes {27a}

We will conduct intent-to-treat analysis. All participants who were originally assigned to different treatment conditions are analyzed as part of their original conditions, regardless of whether they completed the treatment or adhered to the protocol. Analyses will be performed using SAS or R software. All statistical tests will be two-tailed (*α* = 0.05). The treatment condition will be compared to the control condition; if significant differences are observed (*p* < 0.05), the results will be interpreted as evidence of treatment effect.

Descriptive statistics for all measurements will be reported separately for each trial (people who smoke with SPD, with low SES, and with neither). Frequency distributions will summarize categorical data, and measures of central tendency and dispersion will summarize continuous data. All data will be examined for bivariate relationships with the outcomes and with each other.

For each trial, the primary analysis will be regression models to test the difference in the mean CPD (cigarettes smoked per day) between those in the treatment condition and those in the control condition, adjusting for the baseline CPD value and other covariates that are strongly correlated with the outcome (*r* ≥ 0.3) [49]. Analyses of secondary outcomes will be similar to the analysis of the primary outcome. We will use multivariable regression models to examine differences between the treatment and the control arm while accounting for potential covariables.

### Who will be included in each analysis {27b}

Following an intent-to-treat principle, all randomized participants will be analyzed in the trial condition to which they were originally assigned (VLNCs and messages vs. VLNCs only), regardless of adherence, exposure to messages, product use, or study discontinuation. Primary and secondary outcomes will be analyzed separately within each of the three groups (participants with SPD, low SES, and neither) so that each participant contributes only to the analysis group corresponding to the population in which they were enrolled.

### How missing data will be handled in the analysis {27c}

Initially, the likelihood of missingness will be checked to see whether it is associated with any of the covariates or outcomes. Also, multiple imputation is a method to deal with missing data, which accounts for the uncertainty associated with missing data. Multiple imputation will be implemented in our statistical software (SAS) under the missing at random (MAR) assumption and provides unbiased and valid estimates of associations based on information from the available data. The method affects the coefficient estimates for variables with missing data and the estimates for other variables with no missing data. We will investigate the nature of our missing data, and if an assumption of missing completely at random (MCAR) is invalid, we will utilize multiple imputation methods.

Complete datasets will be created using the multiple imputation by chained equations method, and results across imputed datasets will be combined using Rubin’s method [50]. Results from this approach will be compared with those that we obtain when we use only observations with complete data. Consistency of results across these approaches will be interpreted as providing stronger evidence for the model results found than in the case of the results depending on the approach used. Results of these additional analyses and interpretations will be reported in final products.

### Methods for additional analyses (e.g., subgroup analyses) {27d}

N/a—This trial does not include prespecified subgroup analyses.

### Interim analyses {28b}

Interim analyses will be conducted for the re-estimation of sample size and will be conducted (blinded to investigators) for providing preliminary results to FDA if rulemaking for reducing nicotine in cigarettes to non-addictive or minimally addictive levels is proposed.

### Protocol and statistical analysis plan {5}

Access to the full protocol, participant-level data, and statistical code will be managed by the PI, Dr. Popova, at Georgia State University. Requests for these materials will be reviewed, and data will be made available when appropriate. All study data will be deposited in the ScholarWorks@Georgia State University institutional repository within 1 year of study completion. The compiled dataset will be publicly accessible through the ScholarWorks@Georgia State University website. Users seeking access must adhere to specific conditions as specified by the ScholarWorks. Redistribution of the data to third parties will not be permitted.

## Oversight and monitoring

### Composition of the coordinating centre and trial steering committee {3d}

PI Popova will be responsible for the overall study conduct, oversight, and leadership, as well as for the protection of human subjects and data and safety monitoring. PI Popova and Co-I Spears will be responsible for the oversight of the recruitment, enrollment, and data collection efforts at GSU. Co-I Giordano will provide primary project oversight for the recruitment, enrollment, and data collection efforts at Grady and Emory. Each project site will have a project coordinator who is responsible for running day-to-day operations. The project coordinator at GSU will be in charge of project support from the graduate research assistants. Co-I Kirpich will manage data analysis activities.

### Composition of the data monitoring committee, its role and reporting structure {28a}

A Data and Safety Monitoring Board (DSMB) with relevant expertise in randomized controlled trials, clinical research ethics, VLNCs, smoking cessation, health disparities research, and biostatistics will be appointed by the PI. These individuals (*n* = 3) will be independent of the study team. Proposed DSMB members will be reviewed and approved by the awarding NIH Division Director or designee prior to their appointment. The DSMB will meet at least annually (and more often if needed), in addition to quarterly and any additional reviews of adverse events as needed.

### Frequency and plans for auditing trial conduct {29}

Site PIs will oversee the adherence to the protocol procedures and ensure consistency among research personnel. The project coordinator at each site will monitor the day-to-day operation of the study. We will obtain IRB approval from all institutions prior to beginning the study. All personnel will be trained in study procedures, human protection issues, and regulatory requirements. This will be conducted via an initial start-up meeting for all research coordinators and site principal investigators. Prior to starting recruitment at the sites, an initiation visit will be conducted. At this visit, all research staff will attend to ensure that they possess adequate knowledge of the protocol and procedures. Standard Operating Procedures for all visit procedures, use of equipment, collection, and entry of data will be reviewed on this visit. Any new staff will undergo training in these areas. To facilitate oversight of and collaboration between the research sites, multiple communication methods will be used, with the study communication and sharing strategies including telephone, web conference calls, email, and Dropbox allowing for frequent monitoring and customized, rapid response support for our research partners.

### Protocol amendments {31}

This study was approved by the GSU institutional review board (protocol H25318) and was registered on ClinicalTrials.gov (NCT06787937). Any required amendments would be reviewed by the institutional review board, and participants would be informed by phone or email if such amendments directly impacted their experience in the study. Registration on the ClinicalTrials.gov will also be updated and reviewed accordingly.

### Dissemination policy {8}

Findings from this study will be disseminated through peer-reviewed journal publications and presentations at national and international conferences. Study results will also be shared with public health agencies, tobacco control organizations, and policymakers to inform regulatory decision-making and communication strategies surrounding nicotine reduction policies. Results will be submitted to the digital archive PubMed Central to ensure broad accessibility to researchers, practitioners, and the public.

## Discussion

This study evaluates the effects of messages about VLNCs and the reduced nicotine policy on cigarette consumption, tobacco use behaviors, and perceptions among people who smoke with SPD, low SES, or neither. To our knowledge, this is the first RCT to assess the impact of a message campaign accompanying the real-world use of VLNCs, a crucial factor in the successful implementation of the FDA’s proposed nicotine reduction policy. Prior VLNC trials have focused on product impact without considering how messaging may shape compliance, risk perceptions, and quit intentions. By integrating VLNCs with a communication strategy, this study will provide insights into how public messaging can support regulatory efforts to reduce smoking prevalence and tobacco-related health disparities. We hypothesize that participants who receive VLNCs alongside messages correcting misperceptions and emphasizing cessation will smoke fewer cigarettes per day than those who receive VLNCs alone. Additionally, we anticipate that exposure to messages will be associated with reduced nicotine dependence, greater forgoing of cigarettes, and increased motivation to quit smoking. Findings from this study will contribute to the scientific literature on the role of communication in tobacco regulatory policy and inform public health strategies aimed at maximizing the impact of the FDA’s nicotine reduction policy.

## Trial status

The original protocol is developed in January 2025 and prepared for submission to BMC trials in June 2025. Recruitment began in September 2025, and data collection is expected to conclude by January 2029.

## Data Availability

The final trial dataset will be available to all coauthors. Records that link each participant to their identifier will be kept locked in secure computer files at the sites coordinating data collection (GSU, Emory, and Grady), and only the PI and the project manager will have access to the list of identifiers.

## Abbreviations

AE: Adverse event
CVD: Cardiovascular disease
CO: Carbon monoxide
DSMB: Data and safety monitoring board
DVT/PE: Deep vein thrombosis/pulmonary embolism
FDA: Food and Drug Administration
GSU: Georgia State University
ICF: Informed consent form
IRB: Institutional review board
MAR: Missing at random
MCAR: Missing completely at random
NCI: National Cancer Institute
NICER: Nicotine intervention and communication for empowering reduction
NNCs: Normal nicotine content cigarettes
NRCs: Nicotine research cigarettes
NRT: Nicotine replacement therapy
OR: Odds ratios
RCT: Randomized controlled trial
REDCap: Research Electronic Data Capture
SAE: Serious adverse event
SES: Socioeconomic status
SPD: Serious psychological distress
VLNC: Very low nicotine cigarette
